# A real-time fMRI neurofeedback system for the clinical alleviation of depression with a subject-independent classification of brain states: A proof of principle study

**DOI:** 10.3389/fnhum.2022.933559

**Published:** 2022-08-25

**Authors:** Jaime A. Pereira, Andreas Ray, Mohit Rana, Claudio Silva, Cesar Salinas, Francisco Zamorano, Martin Irani, Patricia Opazo, Ranganatha Sitaram, Sergio Ruiz

**Affiliations:** ^1^Departamento de Psiquiatría, Facultad de Medicina, Centro Interdisciplinario de Neurociencias, Pontificia Universidad Católica de Chile, Santiago, Chile; ^2^Laboratory for Brain-Machine Interfaces and Neuromodulation, Pontificia Universidad Católica de Chile, Santiago, Chile; ^3^Institute of Medical Psychology and Behavioral Neurobiology, University of Tübingen, Tübingen, Germany; ^4^Unidad de Imágenes Cuantitativas Avanzadas, Departamento de Imágenes, Facultad de Medicina, Clínica Alemana- Universidad del Desarrollo, Santiago, Chile; ^5^Laboratorio de Neurociencia Social y Neuromodulación, Centro de Investigación en Complejidad Social (neuroCICS), Facultad de Gobierno, Universidad del Desarrollo, Santiago, Chile; ^6^Department of Diagnostic Imaging, St. Jude Children's Research Hospital, Memphis, TN, United States

**Keywords:** real-time fMRI, neurofeedback, depression, brain-pattern classification, brain-computer interfaces, support-vector machine, neuromodulation, endogenous neurostimulation

## Abstract

Most clinical neurofeedback studies based on functional magnetic resonance imaging use the patient's own neural activity as feedback. The objective of this study was to create a subject-independent brain state classifier as part of a real-time fMRI neurofeedback (rt-fMRI NF) system that can guide patients with depression in achieving a healthy brain state, and then to examine subsequent clinical changes. In a first step, a brain classifier based on a support vector machine (SVM) was trained from the neural information of happy autobiographical imagery and motor imagery blocks received from a healthy female participant during an MRI session. In the second step, 7 right-handed female patients with mild or moderate depressive symptoms were trained to match their own neural activity with the neural activity corresponding to the “happiness emotional brain state” of the healthy participant. The training (4 training sessions over 2 weeks) was carried out using the rt-fMRI NF system guided by the brain-state classifier we had created. Thus, the informative voxels previously obtained in the first step, using SVM classification and Effect Mapping, were used to classify the Blood-Oxygen-Level Dependent (BOLD) activity of the patients and converted into real-time visual feedback during the neurofeedback training runs. Improvements in the classifier accuracy toward the end of the training were observed in all the patients [Session 4–1 Median = 6.563%; Range = 4.10–27.34; Wilcoxon Test (0), 2-tailed *p* = 0.031]. Clinical improvement also was observed in a blind standardized clinical evaluation [HDRS CE2-1 Median = 7; Range 2 to 15; Wilcoxon Test (0), 2-tailed *p* = 0.016], and in self-report assessments [BDI-II CE2-1 Median = 8; Range 1–15; Wilcoxon Test (0), 2-tailed *p* = 0.031]. In addition, the clinical improvement was still present 10 days after the intervention [BDI-II CE3-2_Median = 0; Range −1 to 2; Wilcoxon Test (0), 2-tailed *p* = 0.50/ HDRS CE3-2 Median = 0; Range −1 to 2; Wilcoxon Test (0), 2-tailed *p* = 0.625]. Although the number of participants needs to be increased and a control group included to confirm these findings, the results suggest a novel option for neural modulation and clinical alleviation in depression using noninvasive stimulation technologies.

## Introduction

Brain-computer interfaces are systems in which a translation of biological neural information into digital information is performed, resulting in different types of artificial outputs. These artificial outputs can be used to replace, restore or improve a motor function (Weiskopf et al., 2004; Daly and Wolpaw, 2008; Shih et al., 2012). They can also be used to feedback neural information to the user (neurofeedback) to guide the participant, whether consciously or not, to reach a specific brain state (Birbaumer et al., 2009, 2013; Weiskopf, 2012; Sulzer et al., 2013; Ruiz et al., 2014; Sitaram et al., 2017).

Brain-computer interfaces (and therefore NF) are composed of 4 main elements. These are the neural signal acquisition module, the signal analysis/translation module, the artificial output, and the participant with whom a learning loop is closed. Given how the neural information is accessed, and the neural features extracted to generate the artificial output, there are several variations of neurofeedback modalities, each with different advantage profiles. Thus, the information needed to develop a neurofeedback system can be originated from the electrical, metabolic or hemodynamic neural information, and it can be extracted invasively or non-invasively.

About 20 years have passed since the first successful implementation of a real-time neurofeedback system based on functional magnetic resonance imaging (rt-fMRI NF) (Linden et al., 2021), a non-invasive neurofeedback system of high spatial resolution (Sitaram et al., 2007, 2011; Weiskopf et al., 2007). Several studies have demonstrated that it is possible to perform neuromodulation via rt-fMRI by extracting the relevant features for feedback—whether from circumscribed brain areas (Caria et al., 2007; Rota et al., 2009; Zotev et al., 2011; Pereira et al., 2015), from the functional connectivity between brain areas, or from complex neuronal networks—in order to explore the brain-behavior correlation underlying the achieved neural modulation (Rota et al., 2011; Ruiz et al., 2011, 2014; Yuan et al., 2014; Ramot et al., 2017), and its potential clinical uses. In fact, preclinical and clinical studies with rt-fMRI NF have been quick to develop, for example on patients with schizophrenia, obsessive compulsive disorder, addiction, autism, stroke, Parkinson's disease or depression (Linden et al., 2012; Buyukturkoglu et al., 2013, 2015; Li et al., 2013; Ruiz et al., 2013; Young et al., 2014; Pereira et al., 2019; Rana et al., 2020; Vargas et al., 2021).

Until now, most rt-fMRI NF studies have relied implicitly on the idea that a patient can learn to modulate their brain activity toward a healthy neural state. However, because of the alteration of brain function inherent in different neurological and psychiatric disorders, psychiatric patients may have difficulties achieving a healthy brain pattern by neurofeedback of their own whole brain activity. In depression, it is common to find abnormal functioning of the emotional network (Li et al., 2018; Ebneabbasi et al., 2021) so that restoration of healthy neural activity may be less effective if guided by the patient's own neural activity. Thus, such guidance would be more effective if it came from the neural information of a healthy person, of an external “tutor” as it were (Ritchey et al., 2011; Williams et al., 2020). The first step, then, in developing such a system is to distinguish as closely as possible the neural activity that is associated with the desired cerebral state of the healthy individual. An evaluation of the whole neural activity based on a multivariate pattern system has proved useful to classify the neural activity corresponding to different complex brain states, such as complex emotions (LaConte et al., 2005, 2007; Mourão-Miranda et al., 2005; Sitaram et al., 2011b; Cortese et al., 2016, 2017; Tsumura et al., 2022). This approach is different from localizationist implementations as it does not require prior assumptions about the functional location of the different cognitive or affective functions to be studied or trained. Rather, it uses the brain as a whole and the data analysis delivers a single solution - considered the best one - to differentiate between different brain states. There are different approaches for multivariate pattern analysis, the use of SVM has been one of the most frequently used (LaConte, 2011; Watanabe et al., 2017; Miller et al., 2020; Weaverdyck et al., 2020; Taschereau-Dumouchel et al., 2021).

Here we present the methodology and proof of concept for the creation of an rt-fMRI NF system based on an independent SVM-based classifier for restoring the neural activity of patients with depressive symptoms. We tested this rt-fMRI NF approach in training 7 patients with depressive symptoms to achieve the brain state associated to “the happiness” of a healthy female participant.

## Experimental protocol

**Figure 1:**
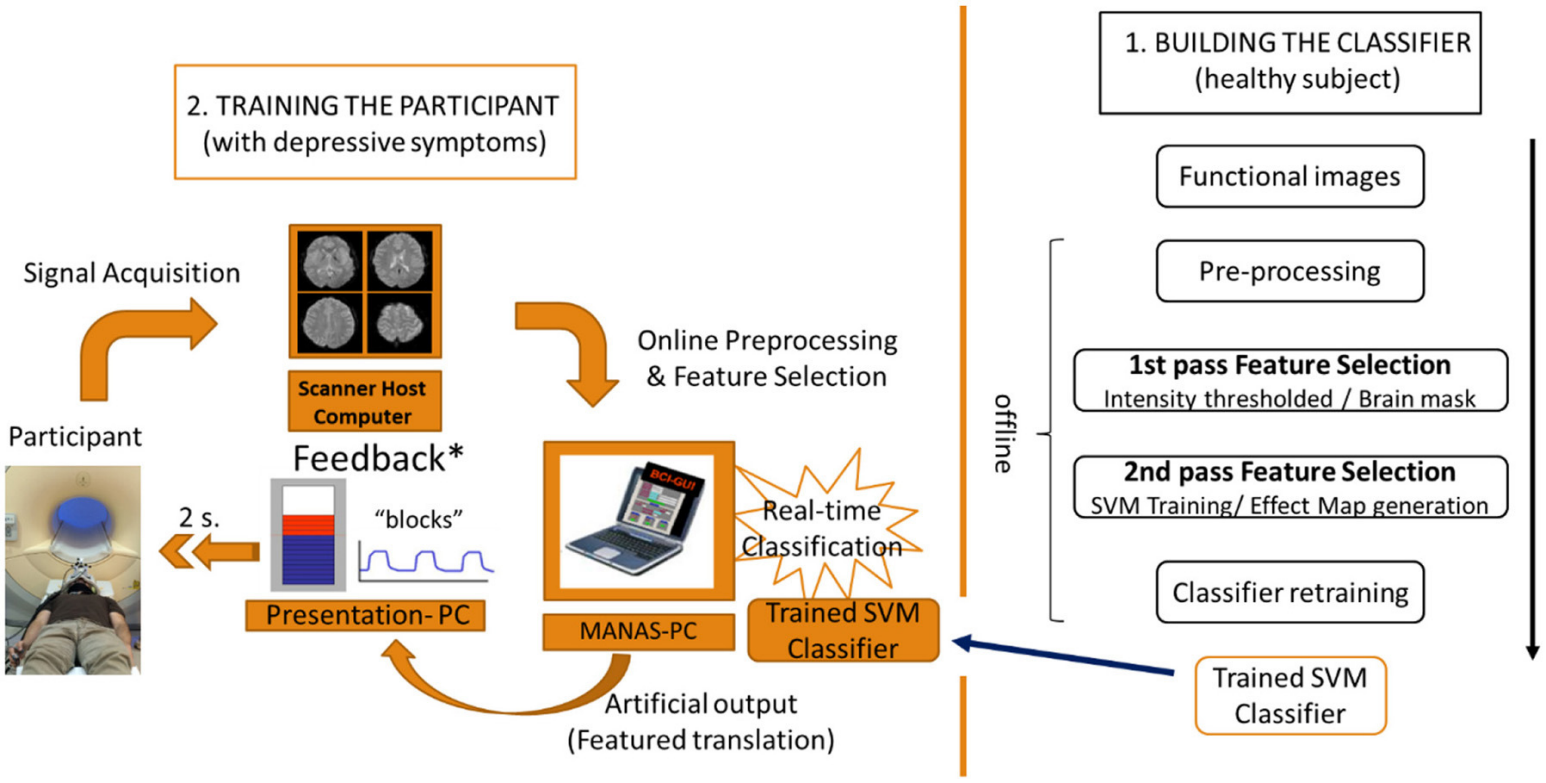
Flow chart of the 2-step experimental protocol. 1. Building the classifier with the neural information (BOLD signal) of the healthy subject. 2. Training of the participant with depressive symptoms using an rt-fMRI NF system based on the classifier.

The methodological discussion is divided into 2 sections. The first section deals with the creation of the brain-state pattern classifier (i.e. happiness versus motor imagery), trained with the information of the whole brain activity of a healthy subject. The second section includes a description of the training protocol, using the rt-fMRI NF system, for the patients with depressive symptoms, making use of the classifier created earlier. The aim of this training is to train the neural activity of a subject with depressive symptoms so that it matches the neural signal features of the healthy subject in a happiness mental state (Figure 1).

### Development of a brain state pattern classifier

In order to distinguish between emotional brain states, a classifier was built as a prerequisite for guiding a patient with depression toward a positive emotional state in the second part of this study. The classifier was based on SVM classification and Effect Mapping of the whole brain of a healthy subject while performing autobiographical memories of happy moments and motor imagery. The use of Effect Mapping was chosen as enhances the classifier based on SVM (Lee et al., 2010) in particular to classifier emotional states (i.e., happiness and disgust), as demonstrated in healthy subjects (Sitaram et al., 2011b).

#### Classifier training

The present study was conducted only with female participants, given that this population has twice the risk of men in presenting depression (Albert, 2015). Thus, the construction of the classifier was obtained from healthy right-handed women without depressive symptoms [i.e. Beck Depressive Symptom Inventory score, second version, BDI-II (Beck et al., 1996) and Hamilton Depression Rating Score, HDRS (Hamilton, 1980), below 7 points on both of them], and without any history of relevant psychiatric or neurological disorders (e.g. bipolar, depression, anxiety disorder, epilepsy, brain traumatic injury or brain tumor) or neurodevelopmental condition (e.g. attention deficit hyperactivity disorder, cognitive deficit, language disorder). A clinical interview was conducted by a psychiatrist to rule out any medical or psychiatric condition.

This research was approved by the Ethics Committee of the Medical Faculty of the Pontificia Universidad Católica de Chile.

#### FMRI data acquisition

A 3T Siemens Magnetom Skyra whole-body MRI scanner (Siemens Medical Solutions, Erlangen, Germany) was used to obtain whole-brain data using a 32-channel head coil. During the fMRI session, the participants were asked to watch the screen specially designed to be used inside the MRI scanner, to remain in the decubitus position comfortably and not moving. First, a Scout sequence to locate and position the planes was conducted at the beginning of the data acquisition. Subsequently, a high-resolution anatomical T1-weighted, three-dimensional MPRAGE sequence (TR/ TE/ TI / flip angle = 2530 ms/ 2.19 ms/ 1100 ms / 7°; field of view = 256; 176 slices, 1 mm^3^ isotropic voxels, 1 mm slice thickness) was acquired for the construction of the classifier and further analysis. In addition, four 2D Echo Planar Imaging (EPI) sequence of 210 volumes and 34 slices, aligned with the anterior and posterior commissure covering the whole brain (TR/ TE/ flip angle = 2000 ms/ 30 ms/ 90°; field of view = 220 mm; 34 slices, 3 mm^3^ isotropic voxels, 3 mm slice thickness) were used to extract the information significant in building the classifier.

#### Block design of functional images

The cognitive task of building the classifier was organized in a block design consisted on 4 repetitions (7 minutes long each run). Each repetition of functional images started with 20 seconds of rest followed by a block (40 seconds long each block) of positive autobiographic memories and a block of motor imagery (i.e. “imagine opening and closing hands palm upwards”), and interspersed with 20 seconds of rest. During the functional sequence participants were asked to look at visual cues on a screen, i.e. “M” (motor imagery), “H” (happiness) or “+” (rest). The participants were asked to train the motor imagery beforehand and to bring a list of 10 happy memories they considered important to them. Between each run they were reminded of the block structure of the experiment, the visual cues to be observed and were also asked about the happy memories they had used in the previous run. All this was done to enssure that during the acquisition of the functional images the participant was performing the cognitive tasks assigned.

#### Offline pre-processing

In order to reduce the noise of the data, the acquired functional images were pre-processed. First, the fMRI DICOM images were converted into NIFTI ones using the Statistical Parametric Mapping Toolbox (University College London) of Matlab (The Mathworks, Inc). The initial 10 functional images were removed in order to delete potential gradient artifact. Functional images were re-aligned to the first volume of the run images and time slice correction was applied. Then, the anatomical scans were co-registered with the mean functional image and segmented. Finally, images were normalized to the Montreal Neurological Institute (MNI) system and normalized using Gaussian kernel (Supplementary Figure 1).

#### Feature selection and classifier training

The development of a classifier of brain states is enhanced by a process of feature selection whereby the informative data is selected to input the classifier training. Previous studies have shown that information selected by Effect Mapping results in better classifiers than the use of other approaches (Lee et al., 2010), mainly when the Effect Mapping is based on a SVM (Sitaram et al., 2011b). SVMs are a type of classifier that maximizes the difference between two (or more) classes of data by computing a hyperplane that separates the two data in the best possible way. In order to train the classifier, the BOLD signals from voxels that belong to the whole brain are extracted by applying an intensity-based thresholding. Linear trends from BOLD signals were removed and informative features from brain signals extracted to create input vectors for training SVM and to obtain the map of effect values over all voxels. Four cross-validations were carried out to test the performance of the classifier in question. Three classifiers and brain masks were developed from three healthy women (18, 26 and 28 years of age) and the classifier with superior classification accuracy was selected for use in the second stage of this study (Table 1). In other words, the same classifier was subsequently used to guide all young adult female patients.

**Table 1:** Offline performance of classifiers in 4 cross-validations.

**Participant** **(years old)**	**Cross-** **validation**	**Accuracy**	**Sensitivity**	**Specificity**
1	1	0.97	0.95	0.99
(28 yo)	2	0.87	0.86	0.88
	3	0.92	0.94	0.90
	4	0.84	0.76	0.93
	Average	0.90	0.88	0.92
2	1	0.90	0.90	0.90
(26 yo)	2	0.96	0.94	0.99
	3	0.96	0.98	0.95
	4	0.91	0.86	0.95
	Average	0.93	0.92	0.95
3	1	0.76	0.80	0.71
(18 yo)	2	0.86	0.89	0.84
	3	0.83	0.81	0.85
	4	0.72	0.75	0.69
	Average	0.79	0.81	0.77

### Real-time subject independent classification and rt-fMRI NF training

In the rt-fMRI NF training the informative voxels previously obtained using Effect Mapping and SVM were used to classify BOLD activity of a second subject (in this case, a patient) in real-time during the neurofeedback run (Effect Maps of these 3 classifiers on Supplementary Figures 2, 3). This classification was converted into real-time visual feedback which was delivered to the patient to guide the neuromodulation process. The aim of the neurofeedback protocol, which consisted of 16 training “runs” performed in 4 days of training over a period of 2 weeks, was to train patients with depressive symptoms so that they could match the brain state associated with happy memories of healthy subjects, in order for the experimenter to evaluate potential clinical changes.

#### Visual feedback and block design of the rt-fMRI NF

The visual feedback was presented to the patients by means of a “thermometer”, with mobile bars refreshed every 2 seconds during the functional images or rt-fMRI NF “runs”. These bars responded contingently to the classification in a binary manner. Thus, one bar was added to the thermometer if the patient's neural information was classified correctly corresponding to the expected brain state (i.e., Effect maps associated either with the motor imagery or with the happiness memory blocks of the healthy subject); in other cases, the bars were subtracted. So, the training runs were divided into blocks, just as the classifier was built with neural information from the healthy subject (Section Offline pre-processing). That is, 4 blocks associated with the happy autobiographic memories of the healthy participants, interspersed with 4 blocks associated with the effect mapping obtained from the motor imagery blocks, and separated by blocks of rest lasting for 20 seconds. During each rest block the thermometer was reset to the mean of the results of the previous block.

In addition to the visual feedback, a monetary reward was used to enhance the learning process, as reported in other studies (Sepulveda et al., 2016). This contingent reinforcement was visually presented in the last 3 seconds of the run and given to them at the end of the 4 runs of each session. The value showed was proportional to the accuracy classification of the classifier on each run. That is, considering a minimum amount of 1.5 USD when the classifier accuracy was 50 percent or less, a linear calculation was performed with increases of 2 USD for every 5 percent increase in classifier accuracy during the run. This is 3.5 USD when the classification results in a value between 51 and 55 percent, 5.5 USD for values between 56 and 60 percent, 7.5 USD for values between 61 and 65 percent and so on).

#### Data acquisition and functional images pre-processing

The MANAS toolbox for Matlab (Mathworks, Inc) developed by our group (Rana et al., 2013) was used to pre-process the functional images in real time, extract the information from them and classify the information using the previously developed classifier. This toolbox has two important features: first, it can normalize (MNI space) the functional images of the patient trained in real time; second, it can incorporate both the classifier and the brain mask previously developed to be used in the online classification. Hence, this toolbox enabled us to perform a rt-fMRI NF based on real-time subject-independent classification (Figure 1).

**Figure 2:**
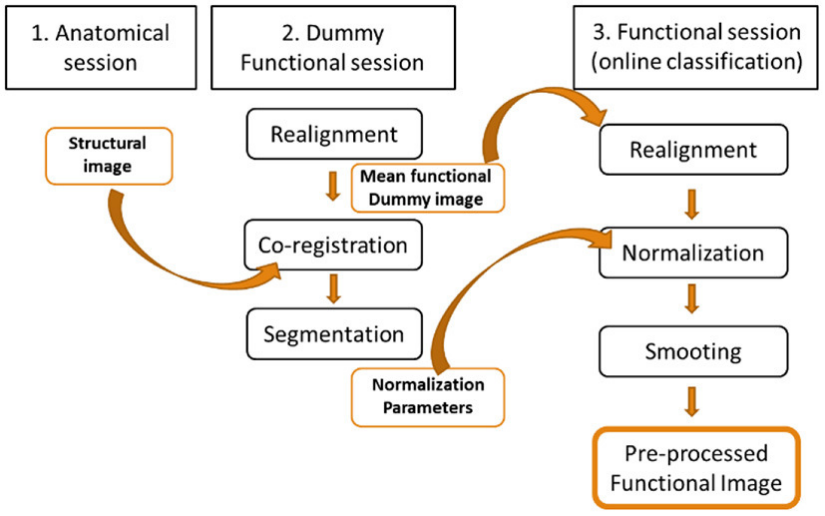
Pre-processing of the functional images for online subject-independent classification. A structural imaging session and a Dummy functional imaging session are performed prior to the functional imaging session of the online classification. The average of the Dummy functional images is co-registered with the structural neuroimaging to obtain the normalization parameters that will be used in the abbreviated pre-processing of the functional neuroimages used in the online classification.

Pre-processing is the first step in performing data analysis of the patient's functional images. It removes as many artifacts as possible for use in online classification. The challenge of real-time classification is to perform all the pre-processing of the functional image before the next one (i.e. 1 TR or 2 seconds in this case). In pre-processing the functional image for subject-independent online classification, the functional images need to be normalized to a standard MNI space for comparison. This requires acquisition of a high-definition anatomical image prior to the functional images, as can be achieved in offline pre-processing. However, co-registration of the functional images with the anatomical images is very time consuming, which makes it impractical to perform in real time. The solution to this problem was to co-register the anatomical image with the average image of a functional EPI sequence identical to those used for online classification, but shorter (i.e. lasting 1 minute). Thus, the normalization parameters for the functional images used in the classification come from the Dummy sequence, significantly reducing analysis time in the online processing. The images were then smoothed using the Gaussian FWHM kernel, finalizing the pre-processing (Figure 2).

#### Feature selection and online SVM classification

To make the analysis more efficient, the most representative voxels were selected from the pre-processed functional images of the patient in real time. The most representative voxels were defined by the brain mask previously made with the functional images of the healthy participant. For the classification process, the information contained in each selected voxel was taken as a vector, and with use of the SVM was compared with the information contained in the corresponding voxel of the healthy participant. The result is a positive or negative classification according to the given condition or block. The classification results were shown to the patient also in real time by means of visual feedback, as previously described. This process was repeated for each functional image, i.e. every 2 seconds for the 7-minute duration of each functional EPI sequence.

#### Recruitment of participants with depressive symptoms

Young right-handed adult women (18 to 31 years of age) with mild to moderate depressive symptoms (with scores of between 10 to 29 points on the BDI-II) were recruited. Participants with severe symptoms (more than 29 points on the BDI-II) or moderate suicidal risk (2 or more points on the suicidality question of the BDI-II or scoring 5 or more points on Okasha's suicidality scale) were excluded and referred to emergency psychiatric care. Participants who were referred to a mental health consultation during the training protocol were excluded from the sample analysis to reduce potential confounders (i.e. clinical changes associated with psychotherapy or antidepressant use). Participants signed the informed consent prior to starting the research protocol.

#### Clinical evaluation

During the day preceding the training sessions (CE1), the day following them (CE2), and 10 days after them (CE3), clinical evaluations of depressive symptoms were carried out using two approaches: a standardized self-report scale (BDI-II) and a standardized clinical evaluation by a certified clinical psychologist (HDRS). In addition, Okasha's suicidality scale (Okasha et al., 1981) was used in case any indicator was observed, either in the BDI-II or in the HDRS, in the questions on suicidality. In the case of scores in excess of 2 points on any one of these three scales, the patient was referred for a psychiatric evaluation on the same day.

### Offline analysis of the rt-fMRI NF

#### Classifier accuracy during the rt-fMRI NF

In order to demonstrate that the binary classification results were not better explained by a random event, a “positive result” had to exceed 50% accuracy (the patient's brain information had to be within the hyperplane corresponding to the desired condition most of the time). Thus, the classifier accuracy of each patient was evaluated for each run (i.e. the quantity of positive classifications according to the condition evaluated/ 210 (total images per Run ^*^100), for each session and for the whole training. Subsequently, both the results for each patient and for the entire group were compared against 50, using non-parametric tests when appropriate.

#### Learning progression during the rt-fMRI NF

The higher the accuracy values of the classification, the greater the similarity of the patient's brain state to the brain state of the healthy participant. Thus, an improvement in accuracy could be considered part of the learning process. Comparisons between the accuracy of the first run and the last run (arithmetic subtraction between Run16-1); between the first two and the last two runs (Run15+16-1-2), and between the mean accuracy of session 4 minus the mean accuracy of session 1 were calculated for each patient. Each of these results was compared against zero to assess the statistical significance of the potential changes resulting from the rt-fMRI NF protocol (non-parametric one-sample Wilcoxon Signed Rank Test compared with zero, p two-tailed, 95% confidence). Slopes across runs and sessions per participant in each group were also obtained.

#### Clinical changes analysis

Potential clinical changes associated with training were assessed immediately after training and 10 days later, using two methods: first, arithmetic subtraction of CE3 and CE1 and subtraction of CE3 and CE2 per patient was performed, and the value was then compared against zero to assess statistical significance. Second, a group pairwise comparison was performed between CE1 and CE2 and between CE2 and CE3, using nonparametric measures for analysis when required.

## Results

### RT-fMRI NF patients

The sample included 7 young adult participants (female, mean age = 25; range = 19 to 31 years old), right-handed, with mild to moderate depressive symptoms (BDI-II: Median = 18; Range = 10 to 29 points/ HDRS: Median = 16; Range = 13 to 19 points), without neurological or psychiatric comorbidity. Participants performed the protocol that involved 4 training days of 4 runs per session/day (over a two-week period), in order to match the selected classifier in both “M” and “H” blocks.

### Classifier accuracy

**Figure 3:**
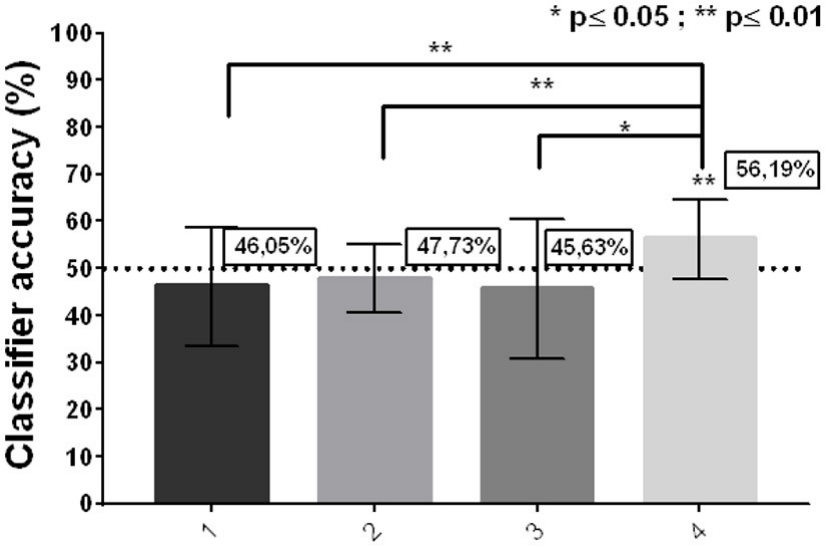
Group classifier accuracy per day. *(Mean* ± *SD; 2-tailed t-test; one-sample z test against zero)*.

Only 2 participants exceeded 50% overall accuracy (average of the 16 Runs per participant = 48.90%; SD: 4.23). However, it was observed that, on the fourth day, 6 of the 7 participants exceeded 50% (Average, Day 1 = 46.05%; SD = 7.99/Average, Day 2 = 47.72%; SD = 4.82/ Average, Day 3 = 45.63%; SD = 10.43/ Average, Day 4 = 56.19%; SD = 6.07; one-sample *z*-test (50) Day 4 p = 0.0035). In addition, a statistically significant difference was observed between the accuracy on day 1 and day 4 (2-tailed *t*-test, p = 0.016), but not between day 1 and day 2 or day 3 (Figure 3).

**Figure 4:**
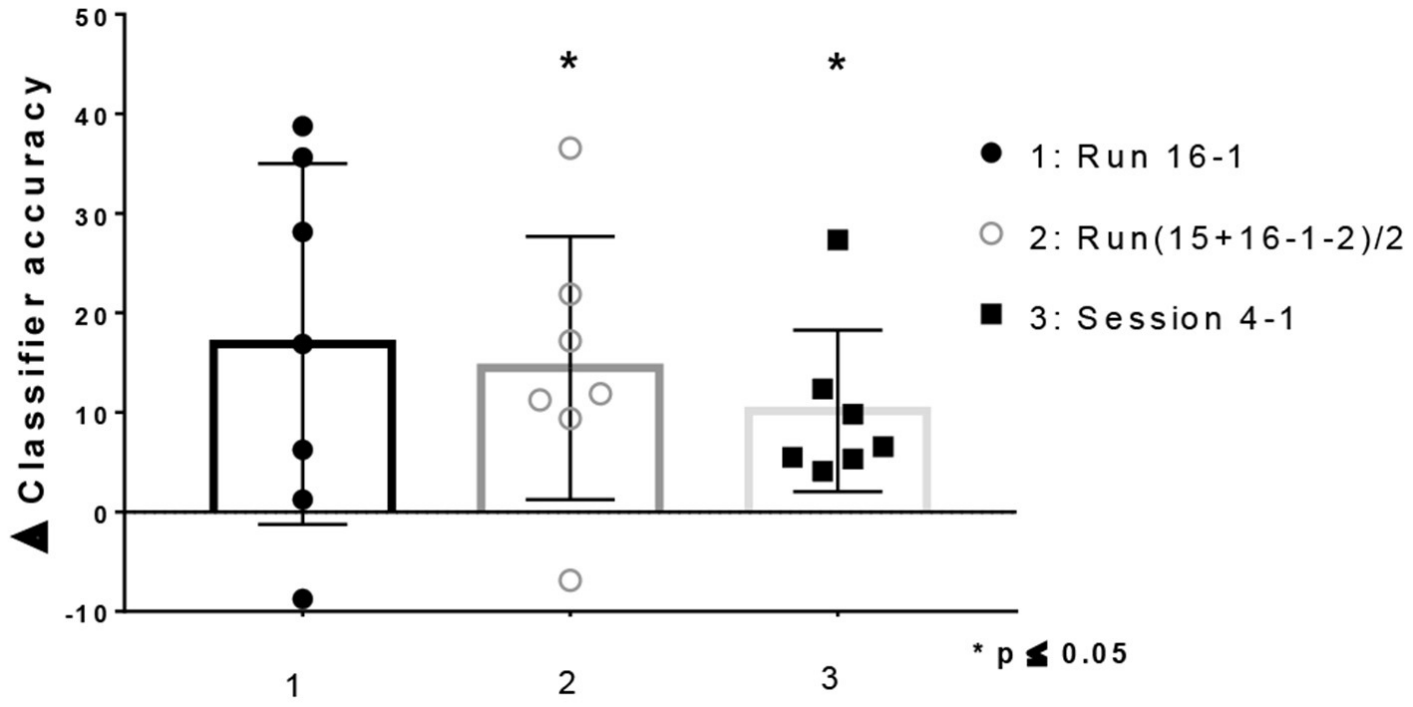
Difference in classifier accuracy between baseline and end of training for patients with depressive symptoms (Mean ± SD. All points plotted. One-sample Wilcoxon Signed Rank Test compared with zero, p two-tailed, 95% confidence).

Improvements in classifier accuracy toward the end of the training were observed in 6 of the 7 patients when comparing the first and last run (Run16–1 Median = 16.88%; Range = −8,75 to 38,75; Wilcoxon Test (0), 2-tailed p = ns), as well as when comparing the last 2 with the first two runs (Run (15+16-1-2)/2 Median = 11.88%; Range = −6.873 to 36.56; Wilcoxon Test (0), 2-tailed *p* = 0.031). Moreover, when comparing by day, improvements in classifier accuracy were observed in all participants [Day4–1 Median = 6.563%; Range = 4.10–27.34; Wilcoxon Test (0), 2-tailed p = 0.031] (Figure 4). On the other hand, all participants showed a positive slope value across runs [Median(R): 0.12; Range: 0.06–0.3] and across training sessions [Median(R): 0.09; Range: 0.02–0.35].

### Clinical changes

**Figure 5:**
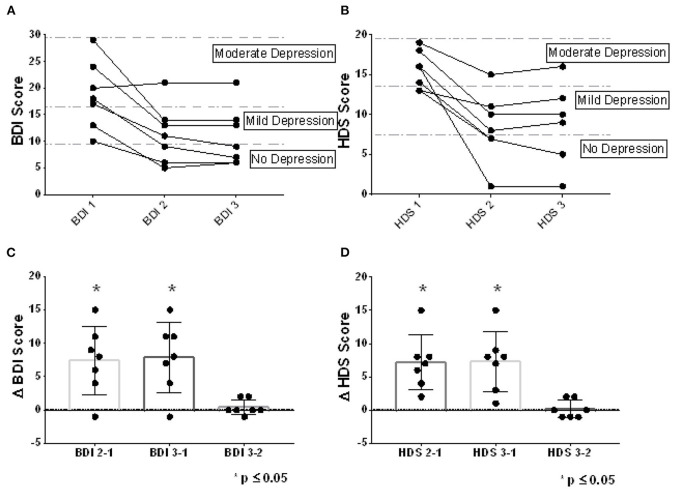
Clinical changes observed. The clinical assessments at baseline, at the end of rt-fMRI NF training, and 10 days later, can be observed for the BDI-II self-report scale **(A)** and for the standardized clinical assessment HDRS **(B)**. In addition, the difference between depressive symptomatology evaluated between baseline, the end of training and 10 days after training, is observed for both BDI-II **(C)** and HDRS **(D)**. The dots represent the assessments for each patient (All points plotted. Mean ± SD. *p ≤ 0.05; One-sample Wilcoxon Signed Rank Test compared with zero, p two-tailed, 95% confidence).

Clinical improvements were observed at the end of the intervention for all participants, either in the self-report assessment [BDI-II CE2-1 Median = 8; Range −1 to 15; Wilcoxon Test (0), 2-tailed *p* = 0.031] or the standardized observation by the clinical psychologist (HDRS CE2-1 Median = 7; Range 2 to 15; Wilcoxon Test (0), 2-tailed p = 0.016), an improvement that was maintained without major variation after the 10-day post-intervention follow-up [(BDI-II CE3-2 Median = 0; Range −1 to 2; Wilcoxon Test (0), 2-tailed p = 0.50/ HDRS CE3-2 Median = 0; Range −1 to 2; Wilcoxon Test (0), 2-tailed *p* = 0.625] (Figures 5A,B). Only one patient reported no improvement at the end of the training (BDI-II), although a favorable change was found in the standardized clinical observation (HDRS). Notably, clinical improvements led to a change from moderate depression to mild depression or from mild depression to “no depression” in most participants (6 participants in the BDI-II measurement and 5 participants in the HDRS assessment) (Figures 5C,D).

## Discussion

A number of rtfMRI NF studies have been attempted in depression. They have been based on our knowledge of the pathophysiology of depression to decide the region-of-interest for self-regulation. Amygdala has been a common area for brain up- and down- regulation (Brühl et al., 2014; Paret et al., 2014, 2018; Young et al., 2014; Herwig et al., 2019) along with other emotion processing areas (Linden et al., 2012; Hamilton et al., 2016; Mehler et al., 2018). A few other studies on depression have attempted to train self-regulation of functional connectivity between brain regions with neurofeedback (Yamada et al., 2017; Zahn et al., 2019). All these studies have used the neural information from the patients to guide the training of their own brain activity. However, if you prepare a pattern classifier from a dysfunctional network, you might train the individual to reproduce the dysfunctional activity due to pattern feedback. Due to the subjects with depression present an altered emotional neural network, we hypothesized that rt-fMRI NF training would be more effective - at both the neural and clinical levels- by using information extracted from a healthy brain as a guide. The aim of this study was to create a subject-independent brain state classifier using information from whole brain as part of an rt-fMRI NF system that can guide patients with depression in achieving a healthy brain state, and then to examine subsequent clinical changes. To our knowledge, this is the first study to use an emotional state classification system developed with information from a healthy third party to guide the neuromodulation process of a subject with depression. On the other hand, several machine learning techniques have been studied for diagnosis and treatment prognosis in depression where SVM has been the most commonly used classification approach [for a review see: (Gao et al., 2018)]. Thus, designing a machine-learning classifier based on SVM seemed to be the best option to elaborate a robust classification and obtain better results at both the neural and clinical levels.

For these reasons we designed a 2-step protocol as a proof of concept. In a first step, a brain classifier based on a SVM was trained from the neural information of happy autobiographical imagery and motor imagery blocks received from a healthy female participant during an MRI session. In the second step, 7 right-handed female patients with mild or moderate depressive symptoms were trained to match their own neural activity with the neural activity corresponding to the “happiness emotional brain state” of the healthy participant. The training (4 training sessions over 2 weeks) was carried out using the rt-fMRI NF system guided by the brain-state classifier we had created. Thus, the informative voxels previously obtained in the first step, using SVM classification and Effect Mapping, were used to classify the BOLD activity of the patients and converted into real-time visual feedback during the neurofeedback training runs. In the protocol used here, the 7 female patients completed the 2-week training, at the end of which they reported symptomatologic improvements that were also observed by a trained clinician.

Certainly, a control group will be needed to fully assess the impact of the rtfMRI NF training intervention on the clinical changes observed, to control for what could be caused by a process of repeated mental imaging of past autobiographical memories by itself. In any case, the clinical results of standard psychological therapies usually take up to 3- 4 weeks to be noticeable (Driessen and Hollon, 2010), an interval during which the neurofeedback intervention presented here could serve as a possible co-adjuvant therapy. Moreover, other neuromodulation techniques based on the patient's own information, such as previous EEG-based neurofeedback systems and rt-fMRI NF, may require longer processes to provide clinical benefits (Peeters et al., 2014). The rapid clinical improvement seen with the protocol described here could be explained, at least in part, by the use of a “tutor” healthy brain as a source of feedback. It is, the feedback obtained from the healthy subject's template that guides the experimental subject's neuromodulation process to a different state than the basal (depressive) one. Another possible explanation for the rapid clinical changes recorded could be due to the use as feedback in this protocol of complex neural networks rather than circumscribed brain areas, as in earlier rt-fMRI NF studies on depression and other psychiatric disorders (Linden et al., 2012; Buyukturkoglu et al., 2013, 2015; Li et al., 2013; Ruiz et al., 2013; Young et al., 2014; Pereira et al., 2019; Rana et al., 2020; Vargas et al., 2021). Thus, to isolate the effect of NF training and to demonstrate clinical effectiveness, it would be necessary to include a control group of matched participants receiving non-contingent feedback (e.g. sham or yolked feedback). It is also possible then to compare this protocol with other rt-fMRI NF systems [e.g., region-of-interest (ROI) neurofeedback], to systematically analyze the pros and cons of each approach. It is still unclear, however, whether the observed clinical benefits would be maintained over time, given that the follow-up at which the clinical improvements were noted was performed only 10 days after the intervention. It has been suggested that a longer period of training could be associated with longer-lasting benefits (Linden, 2014; Tursic et al., 2020).

Regarding the classification accuracy results, these were “not consistent” at the beginning of the rt-fMRI NF protocol and they improved throughout the training sessions. Thus, the classification was clearly due to non-random factors (and probably to the training) toward the end of the training protocol, which further suggests the need to explore protocols with longer training sessions.

On the other hand, it should be noted that here we used a population of the same sex and similar age between those who provided the neural information to develop the classifier and those who participated in the rt-fMRI NF protocol. It will be a matter for other studies to confirm whether this approach is valuable or whether subjects of different sexes or ages can be used. The same applies for different populations. For example, it may be better for adolescent depressive patients to be trained with data from healthy young adults to guide healthy development rather than using data from adolescents of the same age. In addition, another question might be whether a depressive patient with atypical development (e.g. Autism) needs to be trained with information from a typically developing subject without depression or whether it would be more effective to be trained with information from another subject within the autism spectrum without a mood comorbidity. These questions are open for future research.

In summary, this study shows that it is possible to use a subject-independent classifier for depression that potentially leads to clinical improvements, although further confirmation studies are needed. Other neural stimulation techniques, such as transcranial magnetic stimulation and deep brain stimulation have been found to have positive effects in the treatment of anxiety and depression, but disadvantages include potential side effects and invasiveness (Rossi et al., 2020). The results presented here open the door for further exploration of a new clinical approach involving “endogenous neuronal modulation” guided by neuroimages in the treatment of depression, one of the world's most burdensome disorders.

## Data availability statement

The raw data supporting the conclusions of this article will be made available by the authors, without undue reservation.

## Ethics statement

The studies involving human participants were reviewed and approved by the Ethics Committee of the Medical Faculty of the Pontificia Universidad Católica de Chile. The patients/participants provided their written informed consent to participate in this study.

## Author contributions

JP, SR, and RS conceived and planned the experiments and contributed to the interpretation of the results. JP, CSi, CSa, and PO carried out the experiments which were conducted since December 2019. JP, AR, MI, and MR processed the experimental data and performed the analysis. RS verified the analytical methods. JP was responsible for the preparation and writing of the manuscript, which was carried out in collaboration with SR and RS. JP, AR, MR, CSi, CSa, FZ, MI, PO, RS, and SR provided critical feedback and helped to shape the research, analysis, and manuscript. All authors contributed to the article and approved the submitted version.

## Funding

This study was supported by the Comisión Nacional de Investigación Científica y Tecnológica de Chile (Conicyt), through Fondo Nacional de Desarrollo Científico y Tecnológico, Fondecyt Postdoctoral grant (No. 3100648), Fondecyt Regular (Project Nos. 1171313, 117132, and 1211510), and CONICYT PIA/Anillo de Investigación en Ciencia y Tecnología ACT172121.

## Conflict of interest

The authors declare that the research was conducted in the absence of any commercial or financial relationships that could be construed as a potential conflict of interest.

## Publisher's note

All claims expressed in this article are solely those of the authors and do not necessarily represent those of their affiliated organizations, or those of the publisher, the editors and the reviewers. Any product that may be evaluated in this article, or claim that may be made by its manufacturer, is not guaranteed or endorsed by the publisher.

## Abbreviations

rt-fMRI NF: Neurofeedback based on real-time functional magnetic resonance imaging
SVM: Support vector machine
BOLD: Blood-oxygen-level dependent
NF: Neurofeedback
BDI-II: Beck depressive symptom inventory
HDRS: Hamilton depression rating score
MNI: Montreal neurological institute
USD: US dollar
EPI: Echo planar imaging sequence
FWHM: Full width at half maximum
CE: Clinical evaluation.
