# Supplementary figures and images for: A real-time fMRI neurofeedback system for the clinical alleviation of depression with a subject-independent classification of brain states: A proof of principle study
Source: Front Hum Neurosci. 2022 Aug 25;16:933559. doi: 10.3389/fnhum.2022.933559 (PMC9452730; doi:10.3389/fnhum.2022.933559)

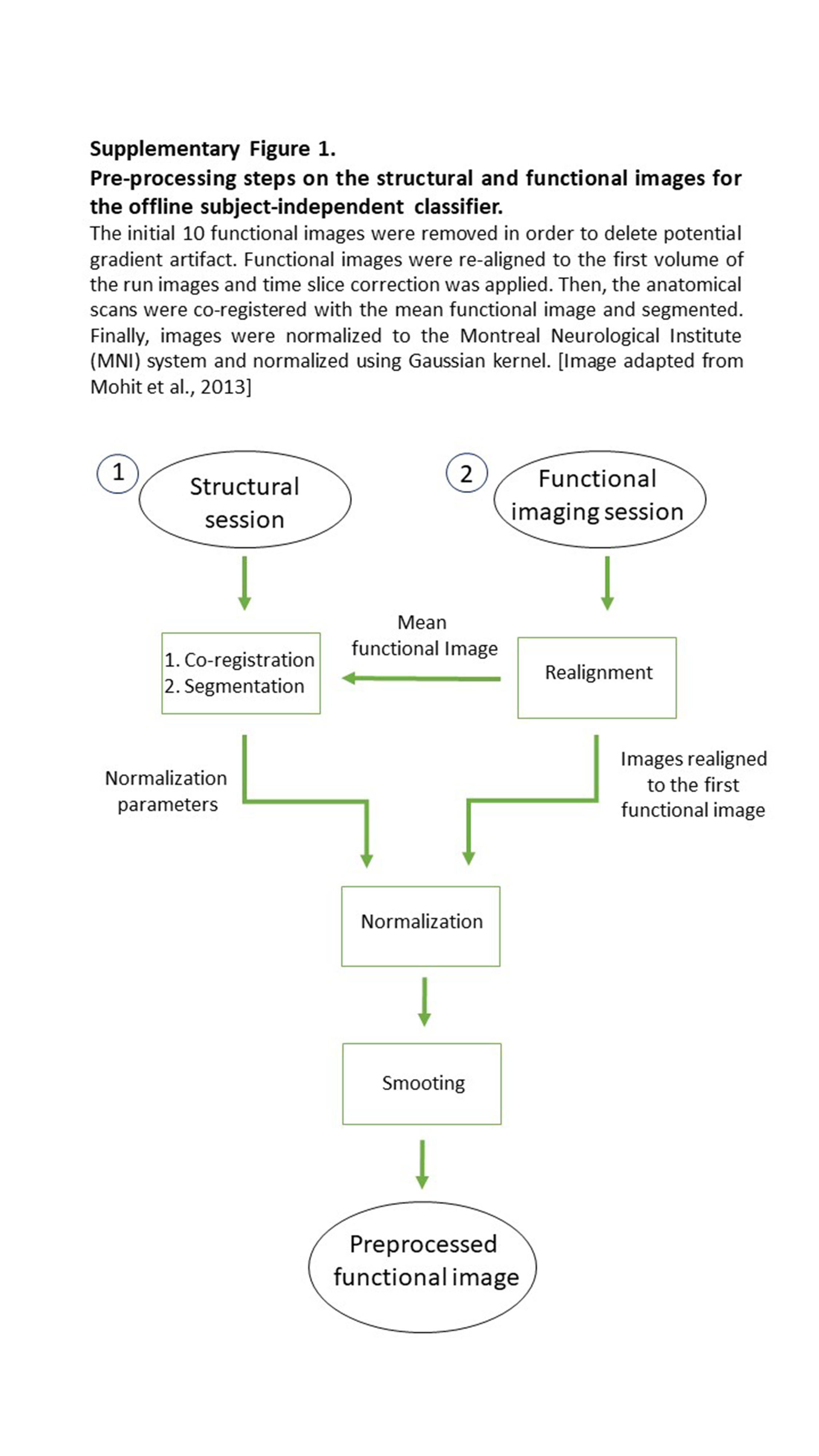

Supplement: Supplementary file 1 [file Image_1.jpg]

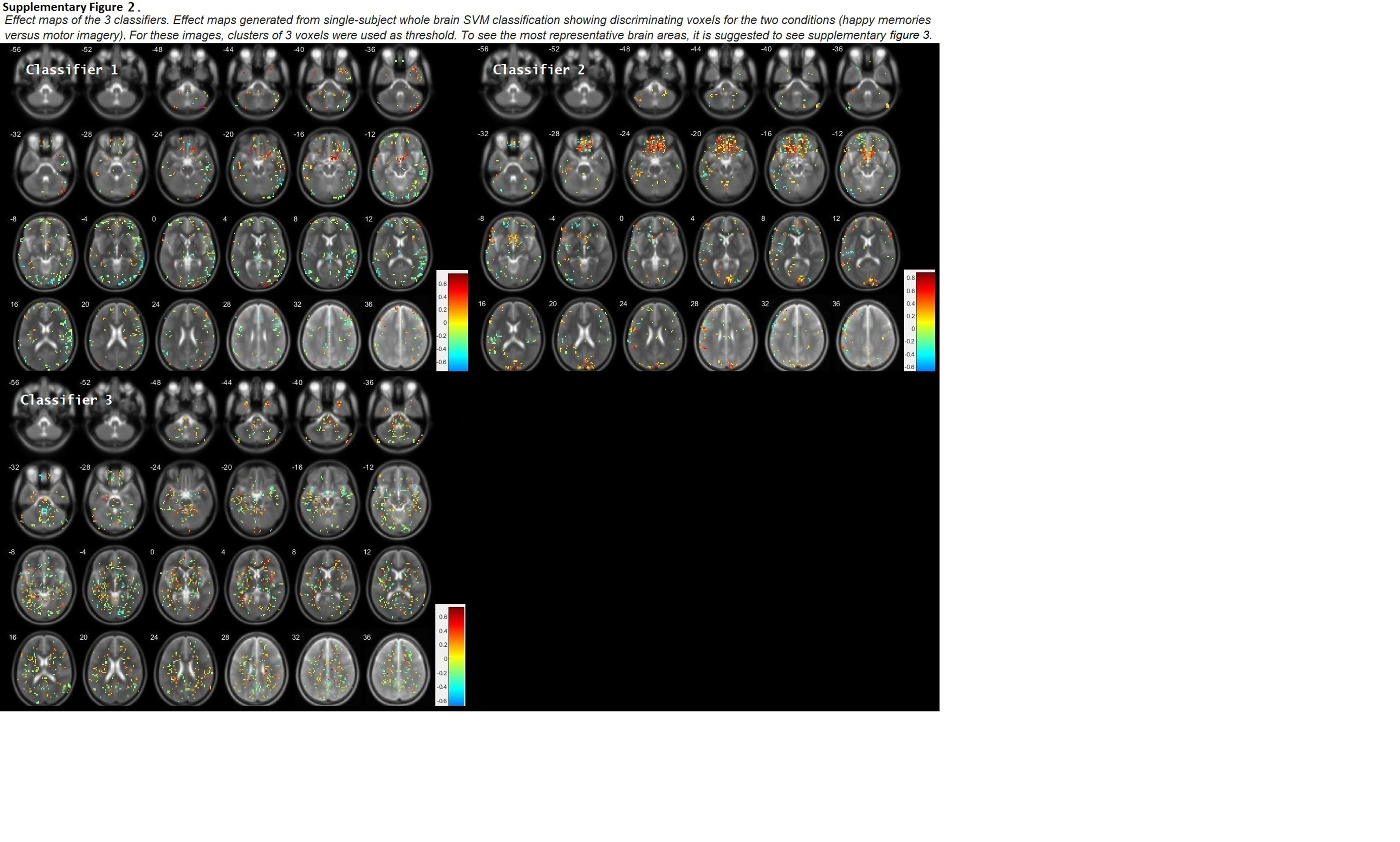

Supplement: Supplementary file 2 [file Image_2.jpg]

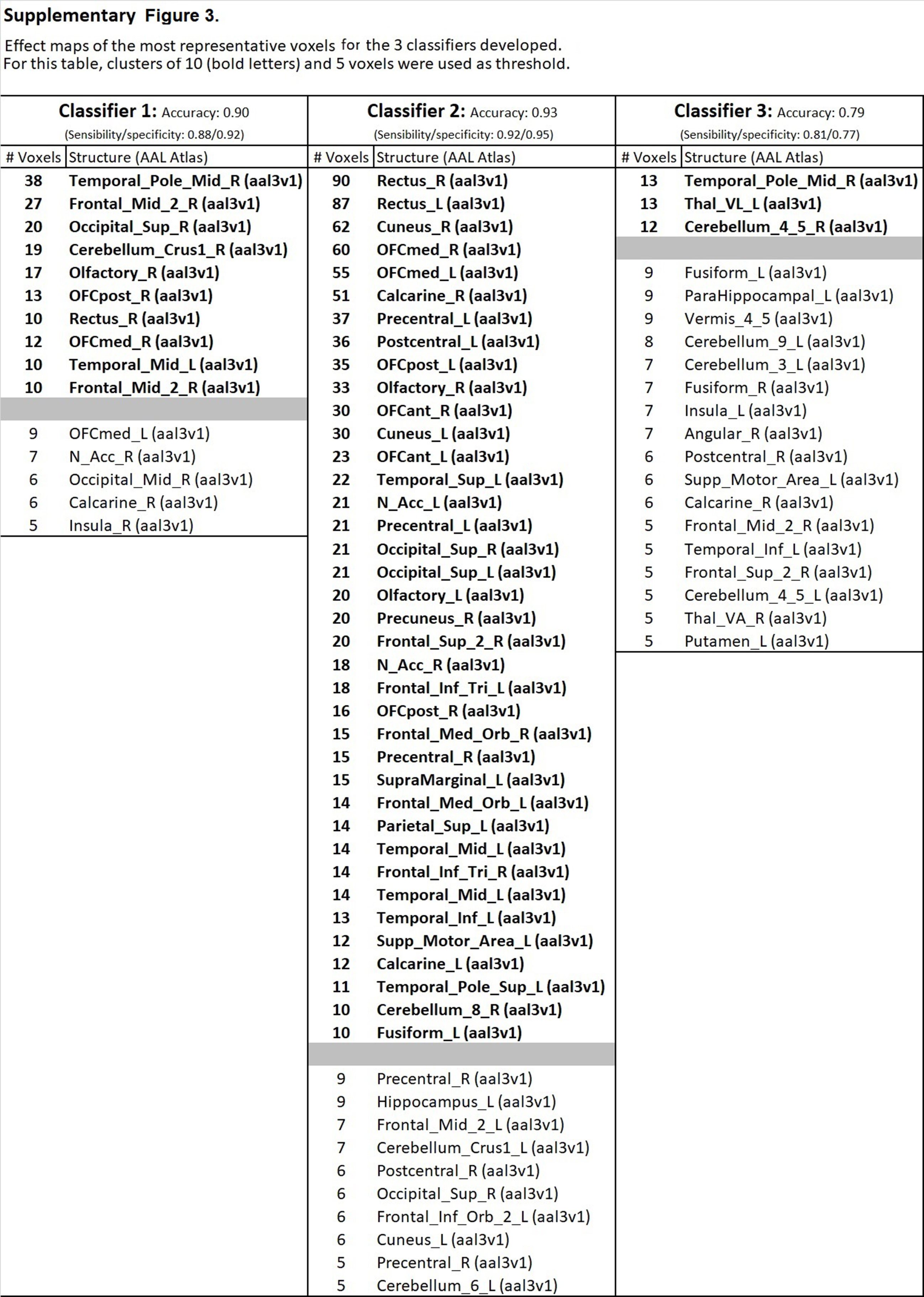

Supplement: Supplementary file 3 [file Image_3.jpg]
